# Femoral strength after cephalomedullary nail removal can be predicted preoperatively using CT based FE models

**DOI:** 10.1038/s41598-025-02424-x

**Published:** 2025-06-06

**Authors:** Alexander Synek, Gilbert M. Schwarz, Andreas G. Reisinger, Stephanie Huber, Sylvia Nürnberger, Lena Hirtler, Jochen G. Hofstaetter, Dieter H. Pahr

**Affiliations:** 1 Institute of Lightweight Design and Structural Biomechanics (E317), TU Wien, Gumpendorfer Straße 7, Vienna, Austria; 2 Division of Trauma-Surgery, Department of Orthopedics and Trauma-Surgery, Medical University of Vienna, Vienna, Austria; 3 Center for Anatomy and Cell Biology, Medical University of Vienna, Vienna, Austria; 4 Division Biomechanics, Karl Landsteiner University of Health Sciences, Krems an der Donau, Austria; 5 Michael-Ogon-Laboratory for Orthopaedic Research, Orthopedic Hospital Vienna Speising, Vienna, Austria; 6 2nd Department, Orthopedic Hospital Vienna, Vienna, Austria

**Keywords:** Finite element, Femur, Cephalomedullary nail, Implant removal, Fracture risk, Biomedical engineering, Trauma, Diagnosis

## Abstract

Removals of cephalomedullary nails (CMNs) after healed pertrochanteric femur fractures are sometimes requested by patients or medically indicated due to pain or screw cut-out. However, CMN removal carries a high risk of secondary femoral neck fracture, even in the absence of trauma. Consequently, decisions on nail removal and establishing a safe post-operative loading regimen can be challenging. This study investigated if finite element (FE) models can pre-operatively predict femoral strength after CMN removal to support these clinical decisions. Nine proximal femora of body donors who were treated with a CMN during their lifetime were included. Computed tomography (CT) scans were acquired with the CMN still in place, followed by virtual implant removal using image processing. Based on this scan, non-linear voxel-based FE models were created and femoral strength was predicted for a one-legged stance configuration. For validation, the CMNs were physically removed and femoral strength was assessed in a material testing machine. The FE models predicted the femoral strength accurately relative to the experiments (*R*^2^ = 0.94, *CCC* = 0.97). In conclusion, CT-based FE models demonstrate potential to predict femoral strength after CMN removal pre-operatively. This could help patients and clinicians to make an informed decision on implant removal and permissible post-operative weight-bearing.

**Supplementary Information:**

The online version contains supplementary material available at 10.1038/s41598-025-02424-x.

## Introduction

Cephalomedullary nails (CMNs) are one of the mainstays in the treatment of pertrochanteric femoral fractures^1^. Despite overall low complication rates, removal of the nail is sometimes requested by the patient or medically indicated due to pain or cut-out of the neck screw^2,3^. However, a high incidence of secondary fractures after nail removal was reported^4^ and biomechanical studies confirmed that nail removal significantly reduces femoral strength^5,6^. This secondary fracture usually happens spontaneously without trauma, is a neck-fracture rather than an pertrochanteric re-fracture and treatment may involve additional surgery with internal fixation or joint replacement^4^. As a result, decisions on CMN removal and permissible post-operative loading regime are still challenging.

To guide these clinical decisions, Barquet et al.^4^ tried to identify general risk factors for secondary fractures after nail removal in a recent review. These include pre-existing systemic osteoporosis, local bone resorption at the femoral neck due to stress shielding, and removal of hardware from the femoral neck. A method that would inherently take all of these risk factors into account is patient-specific finite element (FE) analysis. FE models allow a quantitative prediction of femoral strength, can be created from clinically available computed tomography (CT) scans and take the patient-specific bone shape and distribution of bone density into account^7^. The accuracy of these models was demonstrated in numerous ex vivo validation studies using intact femora^8,9^, femora with local defects^10,11^, and other bones, such as vertebral bodies^12,13^. Given these promising results, they are also increasingly used in clinical studies, e.g. to estimate fracture risk in patients with metastatic bone lesions^14^.

However, to predict femoral strength after removal of a CMN pre-operatively using FE models, several obstacles remain to be overcome that have not yet been addressed in literature. First, the models need to be created from CT scans with the implant still in place, followed by virtual nail removal. This particularly involves the problem of metal artefacts in the CT scan, which can affect the bone and implant shape as well as local bone density^15,16^. Although advances were made in the recent past using dual energy CT scanners, specialized scanning protocols and metal artefact reduction algorithms^17,18^, it remains unclear if FE models can be created from these scans with sufficient accuracy. Secondly, the previously fractured and healed bones may have a highly irregular shape that needs to be captured correctly. Many recent FE models use a smooth representation of the external bone shape^19–21^, but this approach might be unable to capture the geometry of the healed bone or may require substantial manual intervention for the mesh creation. Voxel-based FE models could be a remedy for this issue, as they can capture arbitrary bone shapes, can be easily and automatically created from CT scans, and are only slightly less accurate compared to smooth FE models in studies on intact bone^22–24^. However, their accuracy for predicting femoral strength after a healed fracture and implant removal has not yet been assessed.

The goal of this study was to investigate if non-linear voxel-based FE models can pre-operatively predict femoral strength after CMN removal using a pre-operative CT scan with the implant still in place. Femora with a history of per- or subtrochanteric fracture and treatment with a CMN shall be used to represent a realistic clinical scenario and ex vivo experimental data will be used to assess the accuracy of the predictions. Finally, the predicted femoral strength shall be translated to safety factors for different activities of daily living, to facilitate clinical interpretation of the results and to better guide decisions on implant removal and permissible post-operative loading regime.

## Methods

### Study outline

**Fig. 1:**
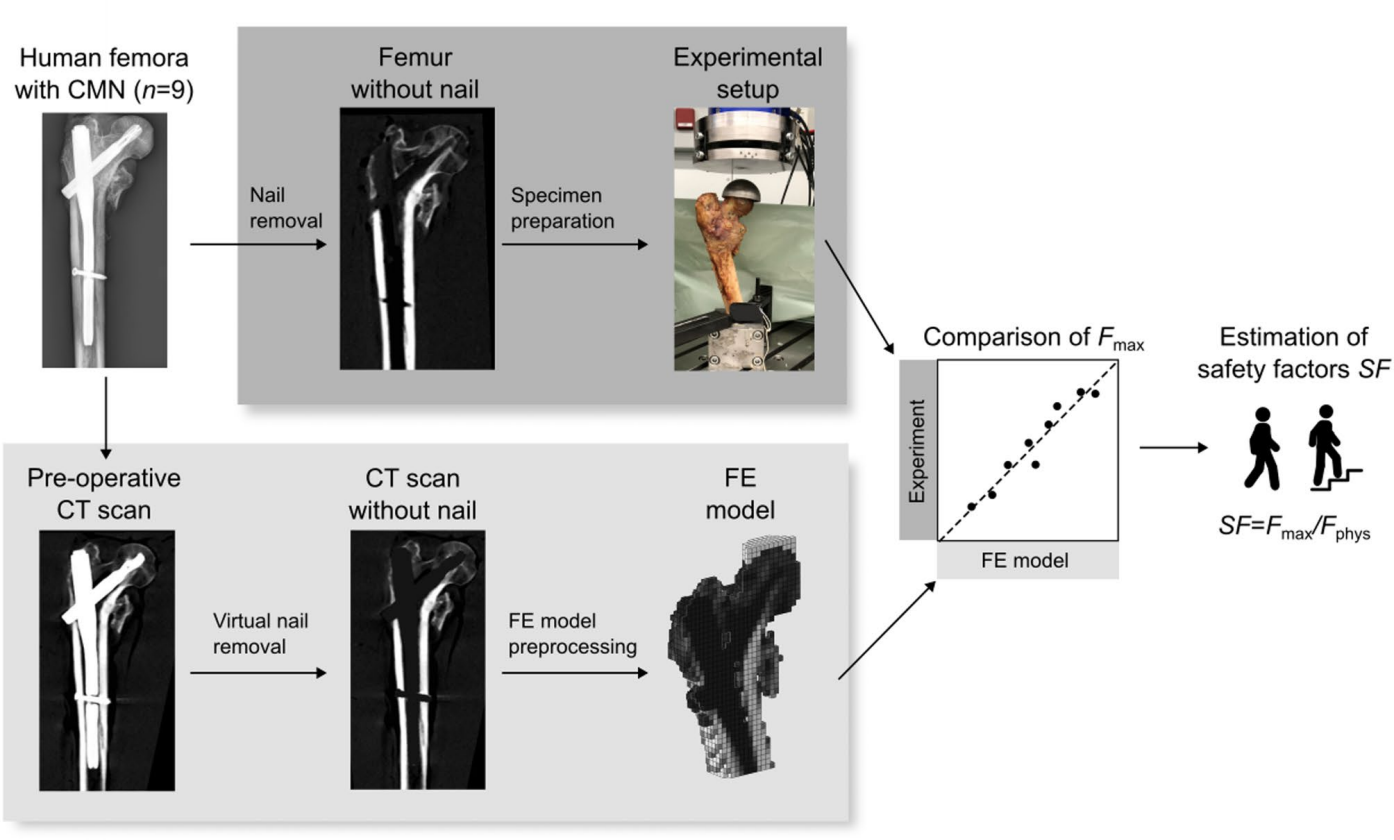
Outline of the study. Nine human proximal femora with implanted cephalomedullary nails (CMN) were used. Femoral strength (*F*_max_) after nail removal in one-legged stance configuration was compared between the experimental measurement and the pre-operative FE model prediction. Safety factors (*SF)* were estimated for clinical interpretation by dividing *F*_max_ with the estimated physiological peak force (*F*_phys_) during various activities.

An outline of the study is shown in Fig. 1 and explained briefly in the following. Nine human proximal femora of body donors with a history of fracture and CMN treatment of a previous experimental study^25^ were included. For experimental validation, the nails were removed and each specimen was loaded until failure in a one-legged stance configuration in a material testing machine to obtain the femoral strength (*F*_max_). For the FE analysis, CT scans were taken prior to implant removal, the nail was virtually removed and non-linear, voxel-based FE models were created. The models were loaded until failure and the femoral strength was assessed. FE model predictions and experimental measurements were compared using linear regression analysis. Finally, the FE-predicted femoral strength was translated to safety factors during different daily activities such as walking and stair climbing.

### Study sample

**Fig. 2:**
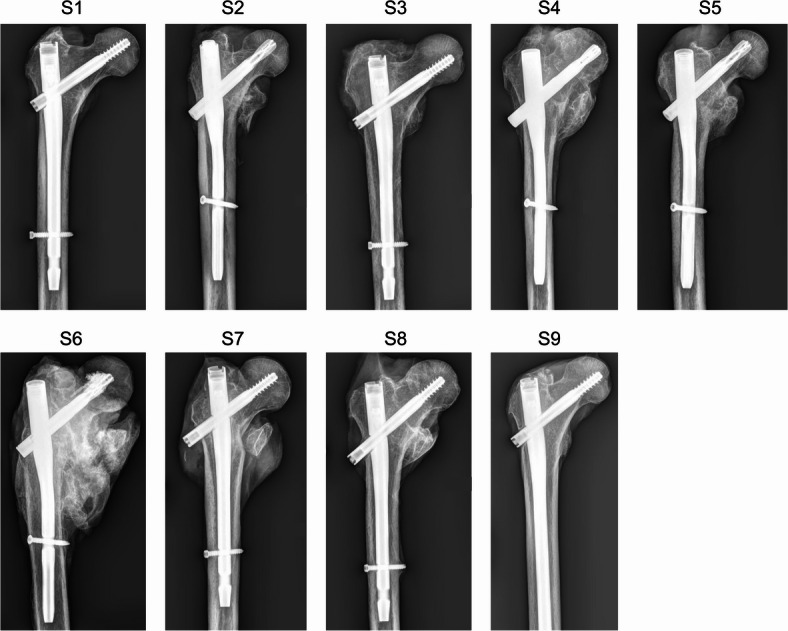
Anterior-posterior radiographs of the nine proximal femora used in this study.

Fresh frozen proximal femora of nine human body donors (Age: 87 ± 8 years; male/female: 1/8; left/right: 8/1) were included (Fig. 2; Table 1)^25^. All body donors had a history of a per- or subtrochanteric fracture and were consequently treated either with a Gamma3 hip fracture nailing system (Stryker^®^, Kalamazoo, USA) or a proximal femoral nail antirotation system (PFNA; Synthes^®^, West Chester, USA) 38 ± 23 months prior to their death. The specimens were obtained from the Vienna Medical Bio-/Implantbank of the Center for Anatomy and Cell Biology of the Medical University of Vienna and all body donors provided informed written consent prior to their death to have their bodies used in medical education and research. The study was ethically approved by the institutional review board of the Medical University of Vienna (EK 2417/2020), and we confirm that all methods were performed in accordance with the relevant guidelines and regulations.

**Table 1:** Age, gender, side, implant type and implant survival of the specimens used in this study.

Specimen	Age	Gender	Side	Implant	Implant survival in months
S1	92	Female	Left	Gamma3	34.8
S2	88	Female	Left	PFNA	9.5
S3	91	Female	Left	Gamma3	86.8
S4	93	Female	Left	PFNA	47.9
S5	86	Female	Left	PFNA	11.1
S6	72	Male	Left	PFNA	44.4
S7	92	Female	Left	Gamma3	42.4
S8	92	Female	Left	Gamma3	39.9
S9	76	Female	Right	Gamma3 long	24.2

### Specimen preparation and experimental testing

Specimen preparation and experimental testing was performed as described in a previous study^25^ (Fig. 1). In brief, the CMN was removed following the manufacturer guidelines, the specimens were cut at the centre of the femoral shaft and embedded both proximally and distally using a polyurethane embedding (SG141/PUR145; FDW Handelsgesellschaft, Austria). The alignment was defined such that the femora were tilted 16° laterally around the anterior-posterior axis and 9° posteriorly around the medio-lateral axes to represent one-legged stance. One-legged stance was chosen as it is considered a particularly critical loading scenario for spontaneous femoral head or neck fractures^26^. The specimens were then mounted to a material testing machine (Z030; Zwick/Roell, Ulm, Germany) by putting the distal embedding in a steel cylinder and covering the proximal embedding with a steel shell. A ball bearing between two steel plates at the proximal end was used to avoid shear forces. The vertical force component was recorded using a multi-axial load cell (Hottinger Baldwin Messtechnik GmbH, Germany). A preload of 10 N was applied, followed by ten load cycles of 100 N. This should ensure that the setup has settled prior to the actual monotonic test until failure. Displacement was then applied at a rate of 10 mm/min until failure was observed, as indicated by a sudden drop of the force. Femoral strength was defined as the maximum recorded force (*F*_max_).

### CT scanning

**Fig. 3:**
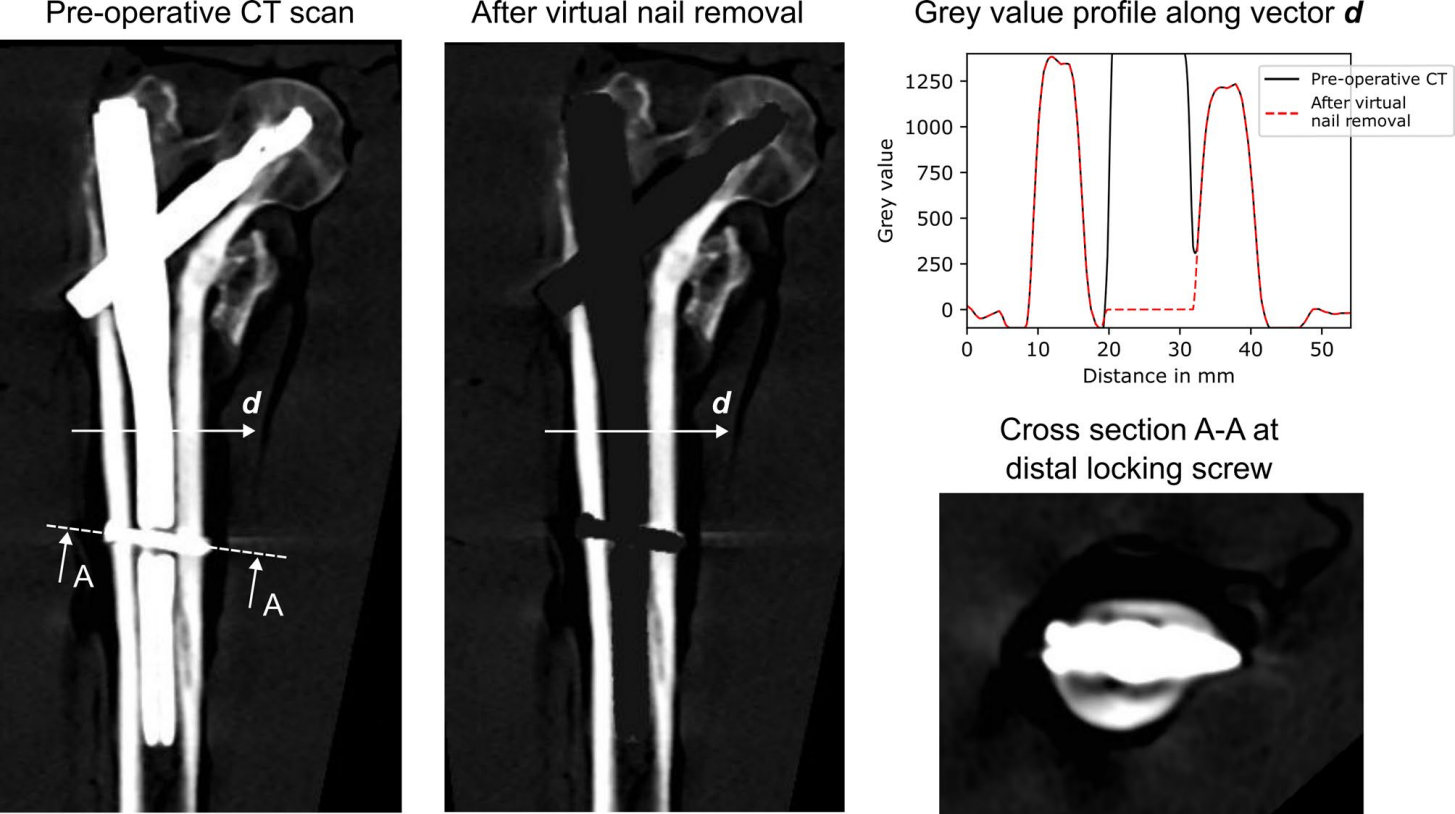
Pre-operative CT scan before and after virtual nail removal for one representative specimen. A grey value profile along vector *d*, and a cross section at the distal locking screw are shown. At the distal locking screw, blooming and streak artefacts were visible.

The pre-operative CT scans were acquired prior to the removal of the nail. The specimens were put in sealed, vacuumed plastic bags and submerged into a container filled with water to mimic the clinical scenario of soft tissue surrounding the bone. A bone mineral density (BMD) calibration phantom (chambers with 0 HU, 100 mgHA/cm³ and 200 mgHA/cm³; QRM, Moehrendorf, Germany) was positioned next to the bone for image calibration. The scans were taken using a SOMATOM Edge Plus scanner (Siemens, Munich, Germany) in dual-energy acquisition mode (tube voltages: 80/140 kV; average tube current: 357/79 mAs). The images were reconstructed with a voxel size of 0.4 × 0.4 × 0.6 mm³ and an iterative metal artefact reduction algorithm of the manufacturer (iMAR; Siemens Healthineers, Munich, Germany) was applied. Metal artefacts in the reconstructed images were moderate except for the region around the distal locking screw, where blooming and streak artefacts around the distal locking screw were visible (Fig. 3). In addition to the pre-operative CT scans, two additional scans were taken from each specimen using the same CT scanning device: One scan was taken after specimen preparation to capture the location of the embedding relative to the bone (tube voltage: 80 kV; average tube current: 25 mAs) and the second scan was taken after testing to identify the fracture location (tube voltage: 140 kV; tube current: 11 mAs).

### Image processing and virtual nail removal

In order to create the FE models from the pre-operative CT scans, several image processing steps had to be performed. First, the nail had to be virtually removed. This was done by creating a mask of the implant and replacing all grey values within this mask with a value of zero. The implant mask was created using single level threshold segmentation and morphological dilation for fine-tuning, i.e. to make sure to remove the implant but not the surrounding bone tissue (Fig. 3). Segmentation threshold and dilation kernel size were manually selected but constant for all specimens. After virtual nail removal, the images were resampled to an isotropic voxel size of 0.5 × 0.5 × 0.5 mm³ and registered to the CT scans taken after specimen preparation to ensure correct alignment with respect to the experimental setup. The specimens were then cropped to 60% of their original height to avoid the influence of remaining metal artefacts around the distal locking screw (Fig. 3), and since neck-fractures rather than shaft fractures were expected^4^. After alignment and cropping, the image processing steps followed the procedure of a previous study on intact femora^8^: The images were linearly calibrated using the BMD calibration phantom with lower and upper cut off values at − 100 and 1400 mgHA/cm^3^, resampled to 3.0 × 3.0 × 3.0 mm^3^ voxel size, masked to include only bone tissue, and additional voxel layers were added at the femoral head to mimic the embedding material. All image processing steps were performed in Medtool 4.5 (Dr. Pahr Ingenieurs e.U., Pfaffstätten, Austria).

### FE modelling

**Fig. 4:**
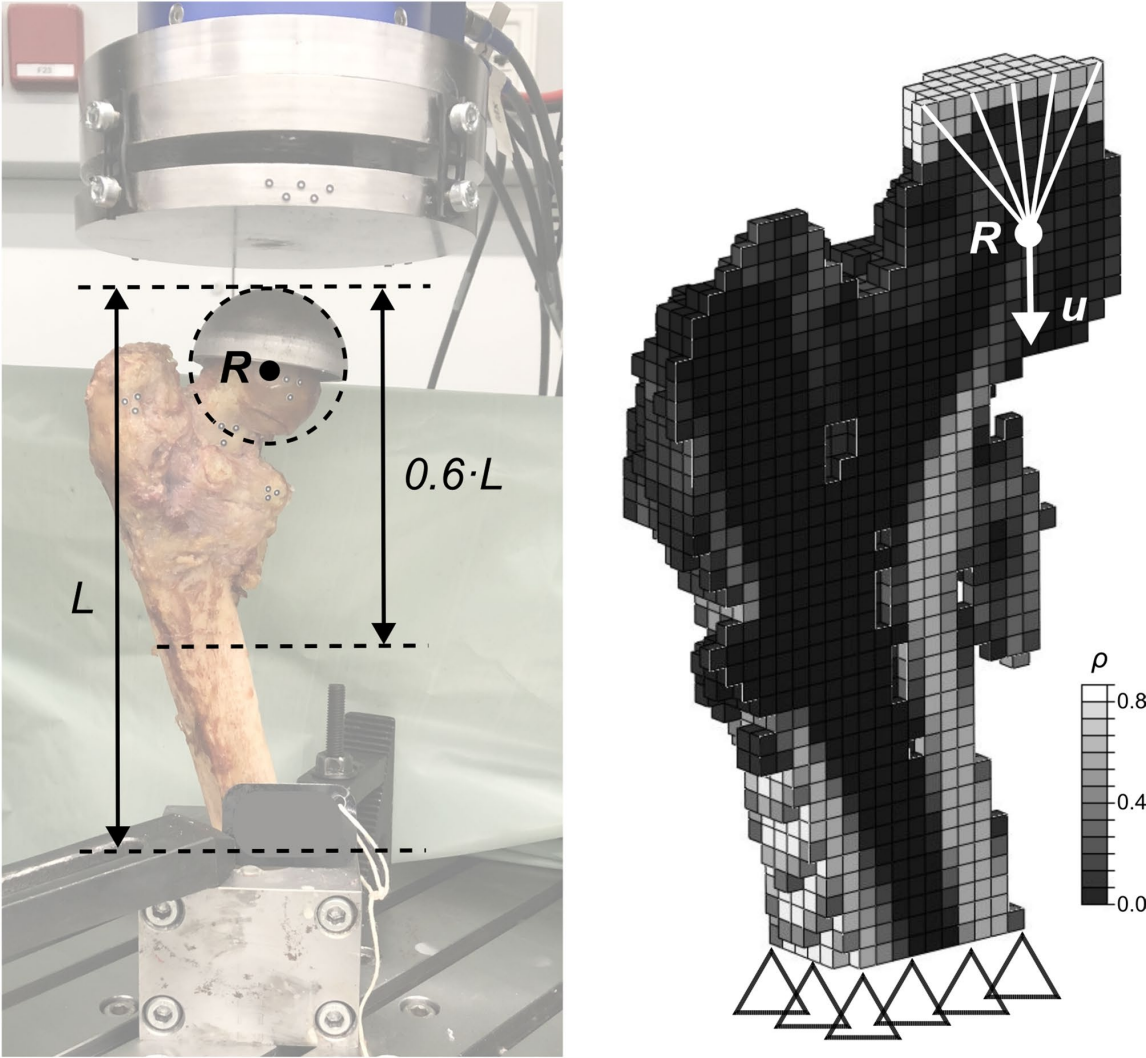
Details of one representative FE model. The model was cropped to 60% of the specimen height *L* and element-specific material properties were assigned based on the bone volume fraction (*ρ*). A reference node was inserted at the centre of a sphere fitted to the proximal embedding. All nodes at the distal end of the femur were constrained and a displacement *u* was imposed on the reference node *R*.

Voxel-based non-linear FE models were created entirely based on the processed images from the pre-operative CT scans. The additional CT scans after specimen preparation were only used to identify embedding locations and alignment in the experimental setup. The models were created following the workflow for conventional CT-based FE models of a previous study^8^ (Fig. 4). Each voxel was converted to a linear hexahedral element with 3 mm side length. This element size was chosen as the original workflow^8^ was developed specifically for this voxel and element size (see Appendix A for a discussion on the element size). The BMD associated with each element was converted to a bone volume fraction (*ρ*) using a linear calibration law. The bone volume fraction of each element served as the input for an isotropic, elastic-damage material constitutive model previously presented by Dall’Ara et al.^8^. In this model, damage is represented by a scalar damage variable *D* which reduces the stiffness (ranging from *D* = 0, up to *D* = 1) and is initiated after reaching the yield surface, which was implemented as a piecewise Hill surface with asymmetric behaviour for compression and tension (see Dall’Ara et al.^8^ for more details). Although this modelling approach led to good correlations to the experimentally measured femoral strength in previous studies, it also quantitatively underestimated the measured values^8,27^. To improve the 1:1 agreement without affecting the correlation, the maximum elastic modulus for cortical bone (*E*_max_) was downscaled from 24,000 MPa to 18,500 MPa while maintaining the maximum yield strength, following an iterative procedure described in Appendix B. *E*_max_ was adjusted as it was only estimated in the original material model^8^, and a value of 18,500 MPa is still well within the range of reported elastic moduli for cortical bone^28^. Since *E*_max_ also drives the extrapolation of the yield constants, they were adapted accordingly to maintain the maximum yield strength of the original material model (see Appendix B). The final material constants are presented in Table 2. Note that the scaling improved the 1:1 agreement of the predictions but only had a very minor influence (< 2%) on their correlation to the experimental results (i.e., the coefficient of determination; see Appendix B). The boundary conditions were defined by fully constrained displacements at the most distal nodes and a uniaxial vertical displacement of 5 mm imposed onto a reference node. This reference node was located at the centre of the sphere used for embedding the femoral head and was coupled to the most proximal nodes of the embedding (Fig. 4). All degrees of freedom of the reference node except for vertical translation were unconstrained. The predicted force-displacement curves were then evaluated and the maximum force was defined as the femoral strength. All pre- and post-processing steps were conducted in Medtool 4.5, and the models were solved using Abaqus 2021HF3 (Dassault Systemes, Vélizy-Villacoublay, France). The models consisted of 8590 ± 3336 elements and the mean time for solving was 120 ± 49 s with 8 CPUs (Intel Xeon E5-2697; Intel Corporation, Santa Clara, USA).

**Table 2:** Final material constants used in this study.

*E*_0_ in MPa	ν	*E*_max_ in MPa	$\:{\sigma\:}_{0}^{+}$ in MPa	$\:{\sigma\:}_{0}^{-}$ in MPa	$\:{\chi\:}_{0}^{+}$	$\:{\chi\:}_{0}^{-}$	$\:{\tau\:}_{0}^{}$ in MPa
6614.0	0.246	18500.0	71.5	95.1	− 0.246	0.333	58.2

For further explanations of the material model, the reader is referred to Dall’Ara et al.^8^. *E*_0_: elastic constant of the trabecular bone material, *ν*: Poisson’s ratio, *E*_max_: extrapolation constant for cortical bone material, $\:{\sigma\:}_{0}^{+}$ / $\:{\sigma\:}_{0}^{-}$: yield strength constant for tension (+) and compression (−) of the trabecular bone material, $\:{\chi\:}_{0}^{+}$/ $\:{\chi\:}_{0}^{-}$: stress interaction constant in tension (+) and compression (−), $\:{\tau\:}_{0}^{}$: shear strength constant of the trabecular bone material.

### Estimation of safety factors

To improve clinical interpretation of the predicted femoral strength values, they were translated to safety factors during various activities of daily living. A safety factor below one indicates that the bone is not strong enough for the respective activity, whereas higher safety factors indicate a greater margin of safety. To estimate this safety factor, the femoral strength *F*_max_ was divided by the peak physiological hip joint force magnitude *F*_phys_ recorded with instrumented prostheses during various activities by Bergmann et al.^29^. In particular, the “AVER75 Average” and “HIGH100 Average” datasets were used^29^: The “AVER75 Average” are peak forces recorded in ten subjects, scaled to a body weight of 75 kg, then averaged, whereas the “HIGH100 Average” are the peak loads of the subject with the highest relative peak loads, scaled to a body weight of 100 kg. The safety factors *SF*_75_ = *F*_max_/*F*_phys, AVER75_ (using the “AVER75 Average” dataset) and *SF*_100_ = *F*_max_/*F*_phys, HIGH100_ (using the “HIGH100 Average” dataset) were then computed for nine different activities (cycling, sit down, stand up, knee bend, walking, one-legged stance, stairs up, stairs down, jogging) as described in Bergmann et al.^29^. A uniform bodyweight had to be assumed for the specimens in this study since the weight of the body donors was not available. Also note that these safety factors are just rough estimates, as they do not take muscle forces or differences in hip joint force vector direction into account.

### Output variables and statistics

The experimentally measured and FE-predicted femoral strengths were compared based on linear regression analysis. The coefficient of determination (*R*²), Lin’s concordance correlation coefficient (*CCC*)^30^ and root mean squared error (*RMSE*) were evaluated. The FE model predictions were considered accurate if the *CCC* reaches at least 0.95, i.e. a “substantial” agreement^31,32^. In addition, the location of the fracture in the experiments was qualitatively compared to the damaged regions in the FE models. As complementary information for further interpretation, von Mises stress distributions were evaluated (see Supplementary Figure S1). Finally, the safety factors were evaluated to investigate their values and variability across body donors. Descriptive statistics are presented as mean ± standard deviation if not denoted differently, and all statistical analyses were conducted in Python using SciPy^33^.

## Results

### Comparison of femoral strength

**Fig. 5:**
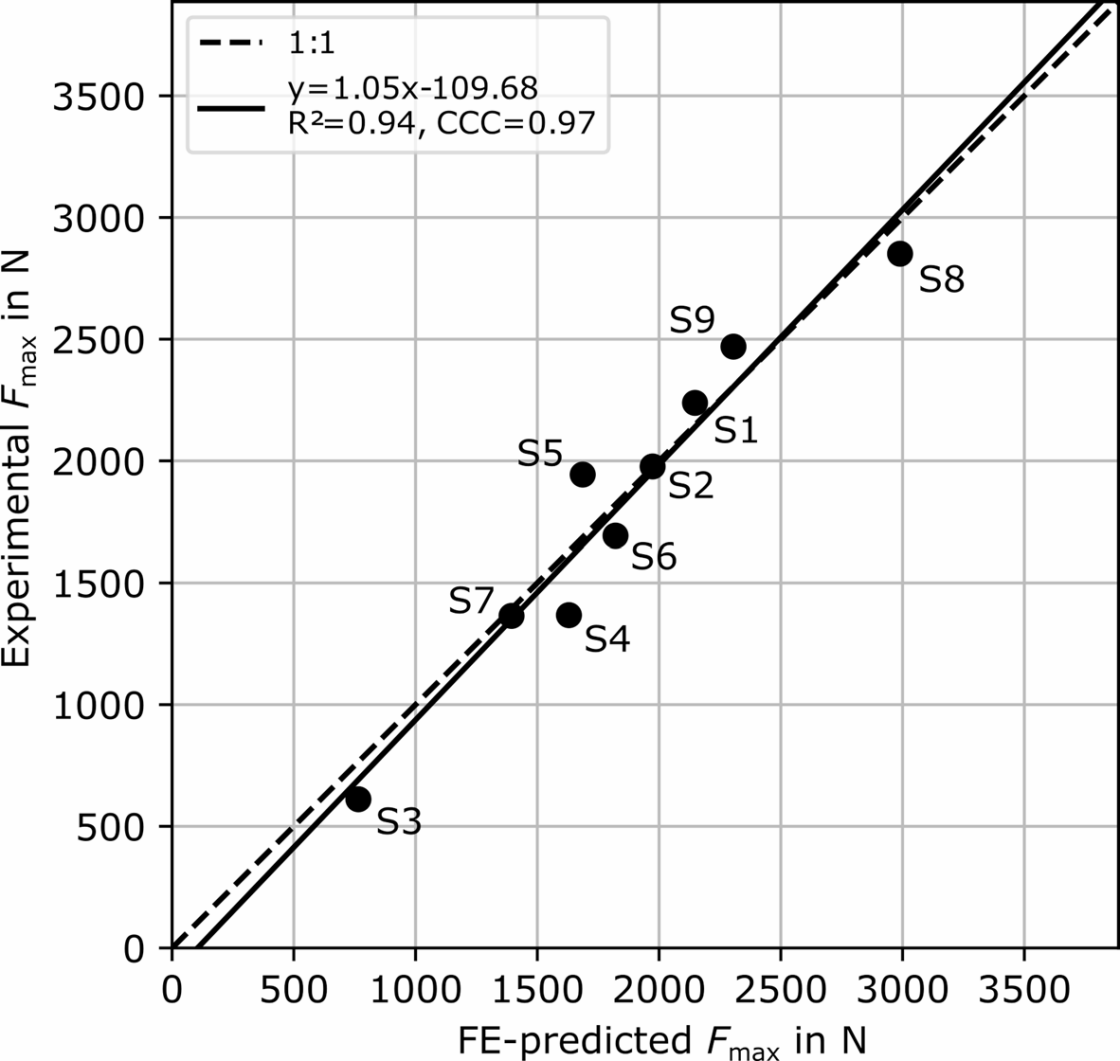
Comparison of experimentally measured and FE-predicted femoral strength (*F*_max_). Coefficient of determination (*R*^2^), concordance correlation coefficient (*CCC*) and the line equation of the linear regression are shown. Labels of individual specimens are displayed next to the data points.

Experimental and FE-predicted femoral strength correlated very well (*R*²=0.94, *p* < 0.001; Fig. 5). The FE model predictions were also in very good quantitative agreement with the experimental measurements (*CCC* = 0.97, *RMSE* = 159.9 N; Experimental *F*_max_=1835.1 ± 631.3 N, FE-predicted: *F*_max_=1857 ± 584.1 N).

### Comparison of fracture locations

**Fig. 6:**
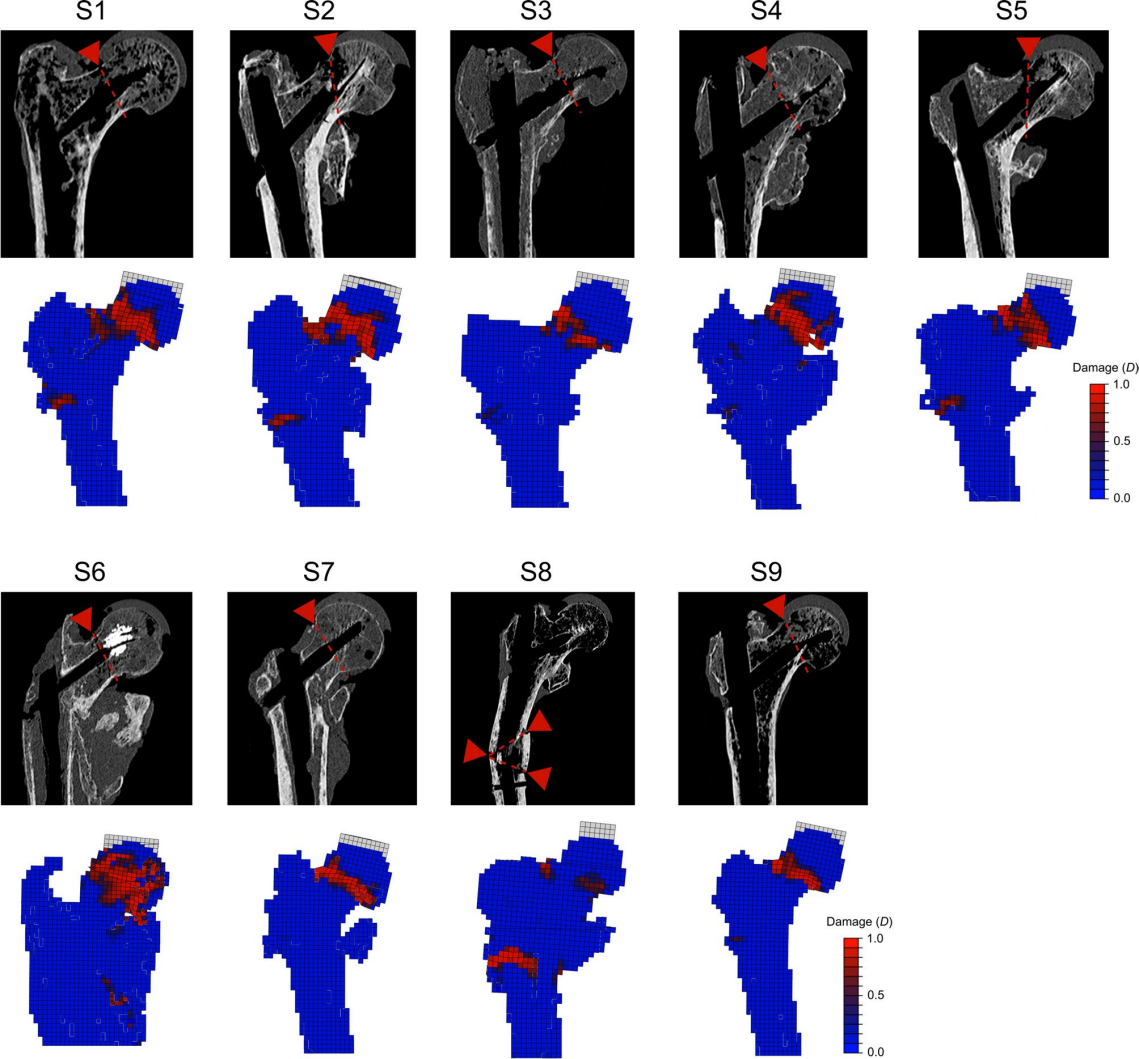
Comparison of the estimated fracture locations observed in the experiments to the damaged regions in the FE models. Fracture locations of the experiments were estimated based on CT scans taken after testing, visual inspection of the specimens, and video recordings. The damaged regions of the FE models are displayed based on a frontal cross section at the last time increment of the simulation.

The fractures observed in the experiments were predominantly located in the femoral neck region; only one specimen (S8) fractured in the shaft region (Fig. 6). These fracture locations were roughly in agreement with highly damaged regions of the FE models (Fig. 6): Specimen S8 showed the highest damage in the shaft region, whereas other specimens showed highest damage in the head-neck region. However, the locations did not match exactly. For instance, a highly damaged region in specimen S8 was located in the shaft region but more proximal compared to the fracture location in the experiments.

### Estimated safety factors

**Fig. 7:**
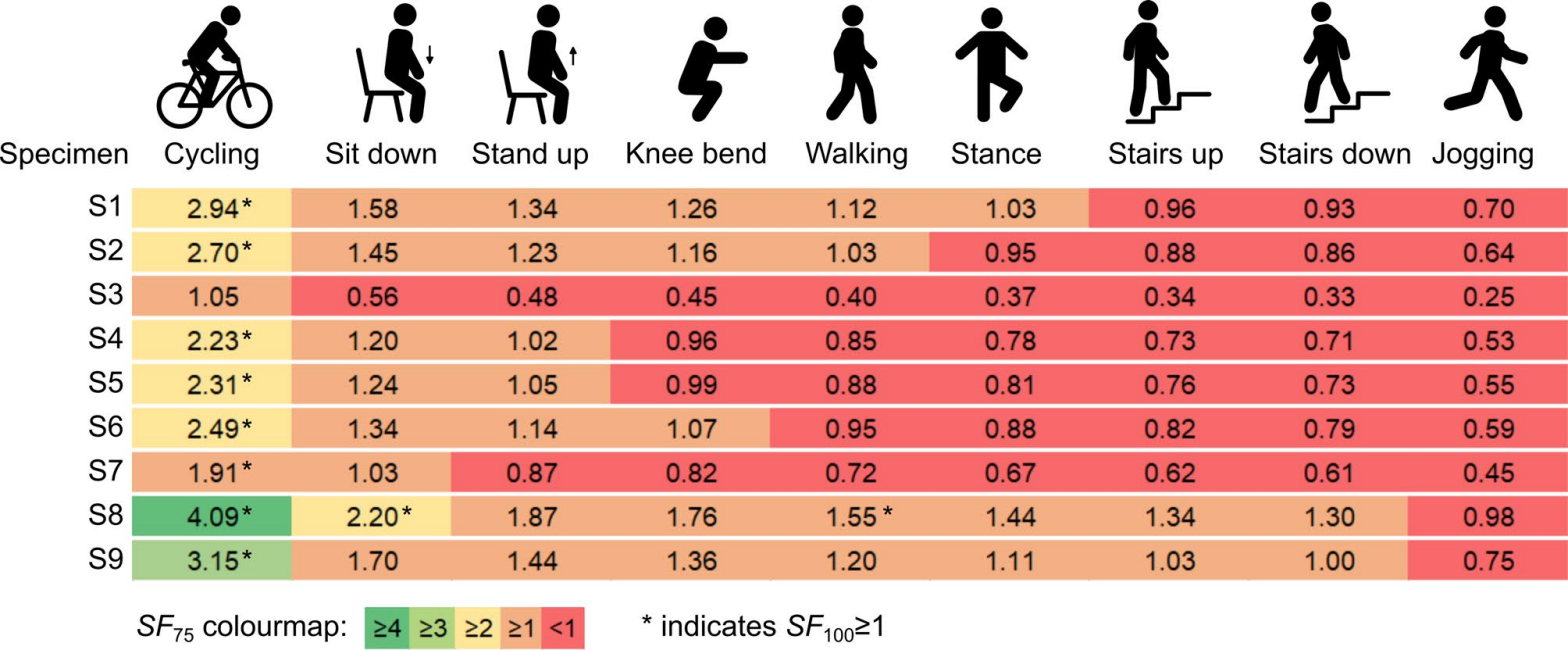
Estimated safety factors *SF*_75_ for all nine femora and different daily activities. Safety factors were computed by dividing the predicted femoral strength of the FE models (*F*_max_) with the averaged in vivo peak hip joint load of subjects scaled to 75 kg body weight (taken from Bergmann et al.^29^). Femora and activities which reached a safety factor *SF*_100_ ≥ 1 are highlighted with * (i.e. using in vivo hip joint loads of the subject with highest relative loads and scaled to 100 kg body weight from Bergmann et al.^29^).

Safety factors were overall low for all specimens and activities (range *SF*_75_: 0.3 to 4.1; range *SF*_100_: 0.2 to 2.4; Fig. 7). Only four specimens reached a safety factor *SF*_75_ of one or higher for walking, and only one also reached *SF*_100_ higher than one for walking. Cycling was associated with the highest safety factors, but the variability was considerable (range *SF*_75_: 1.1 to 4.1; range *SF*_100_: 0.6 to 2.4). Two specimens reached *SF*_75_ of one or higher for all activities except jogging. Only one specimen reached *SF*_100_ of one or higher for any other activity than cycling (a table of all *SF*_100_ values is available in Supplementary Table S1).

## Discussion

The goal of this study was to test if FE models can pre-operatively predict femoral strength after CMN removal in order to provide clinical decision support. It could be shown that voxel-based non-linear FE models can accurately predict femoral strength relative to ex vivo experimental data (*R*²=0.94, *CCC* = 0.97, *RMSE* = 159.9 N), despite the metal implant in the pre-operative CT scan and the highly irregular geometry of the previously fractured and healed bones. Translating the predicted femoral strength to a safety factor for different activities of daily living showed that all specimens would be at high risk of a fracture after nail removal, although the variability among specimens was considerable.

The agreement of FE model predictions and experiments in this study is in line with previous studies on intact femora and femora with other defects (e.g. metastases)^7,34^. The coefficient of determination (*R*²) for femoral strength achieved in this study was 0.94, which falls well within the range reported in literature amounting to 0.35 to 0.96 for intact bones^7^ and 0.47 to 0.98 for femora with metastases^34^. Good quantitative agreement of femoral strength was achieved in some^11,35^ of these studies, whereas over- or underestimation of experimental measurements were observed in others^8,27,36^. Similar to the results presented in this paper, fracture locations in previous studies often, but not always matched with highly damaged or yielding regions^8,37,38^. Also note that the visual identification of the fracture location based on image data is challenging and prone to errors as well. In contrast to previous studies, the material constants were scaled in this study to not only achieve a good correlation, but also a good quantitative fit the experimental data. Note that the coefficient of determination was very high irrespective of this scaling procedure (*R*²>0.9, see Appendix B), and the scaling merely served the purpose of a meaningful interpretation of the predictions. Overall, this study further underlines the good predictive abilities of FE models for femoral strength predictions and extends possible use cases to pre-operative simulations after virtual implant removal.

One major challenge compared to previous CT-based FE models of intact bone was the presence of the metal implant and related artefacts in the CT scan. Particularly the interface between a metal implant and bone may be subject to artefacts^15^ and simulations including both bone and implant may need an adaptation of the modelling workflow (e.g. by adapting the BMD-elasticity relationship in this region^16,39^). When simulating the scenario after implant removal, the interface itself is less relevant for the model predictions. This study showed that standard clinical CT scans are indeed sufficient to create FE models that predict femoral strength after metal implant removal with good accuracy using a standard modelling workflow. However, it also showed limitations due to remaining metal artefacts. For instance, the BMD in the cortex around the distal locking screw erroneously appeared strongly reduced, which introduced an artificial weak spot in the bone. This problem was circumvented in this study by excluding this region from the model, which was acceptable considering that fractures in this region were not expected. Still, the CT scanning protocol should be improved to allow accurate simulations of hardware removal also for the entire bone and implant in the future. A systematic exploration of alternative scan protocols with even higher energy X-rays or using photon-counting detector CTs^18^ may be a way forward to achieve this goal.

To improve the interpretation of the predicted femoral strength values both for patients and clinicians, the predicted femoral strength was translated to safety factors for different daily activities. Overall, the safety factors computed in this study were low compared to safety factors reported for healthy individuals. For instance, estimated *SF*_75_ ranged from 0.3 to 1.6 for walking and stair climbing in this study, whereas Taddei et al.^40^ reported an average safety factor of five based on computational modelling of 200 healthy subjects. Thus, in a clinical scenario, CMN removal would most likely not be recommended without further treatment for any of the femora investigated in this study. This was to be expected given the age of the body donors (average: 87 years) and is in line with the general clinical recommendation to avoid CMN removal whenever possible^4^. However, FE-predicted safety factors may be useful in making decisions on CMN removal in younger patients. The FE models could identify patients with sufficient residual femoral strength after nail removal, avoid unnecessary treatments and even give an outlook on permissible activities during rehabilitation. Still, it must be mentioned that the estimation of safety factors in this study is highly simplified and should be refined prior to clinical use. Specifically, the loading conditions in this study only involve uniaxial force application at the hip joint representing one-legged stance, whereas in reality, hip joint force vector directions vary throughout movements and several muscle forces are acting on the femur^40,41^. A previous study on intact femora showed that the one-legged stance load case is most critical for head or neck fracture predictions and muscle forces may be neglected in this use case^26^, but these findings remain to be confirmed for femora after implant removal. Also, the FE-predicted safety factors as presented in this study can only judge the current situation without taking fatigue failure and future bone (re)modelling into account.

Several limitations of this study must be mentioned. First, the FE model was used to replicate an ex vivo experiment, which has several inherent limitations. For instance, the loading scenario was simplified, soft tissues were not included and the subject age was rather high. Second, ex vivo CT scans were used to create the FE models. Although a clinical scan protocol was used and water around the bone was included to mimic soft tissue, the applicability of the workflow to in vivo CT scans still needs to be investigated. Third, the FE models were limited to the proximal part of the femora in this study to exclude the effect of remaining metal artefacts at the distal locking screw. Although this was considered sufficient for this study, the CT scanning protocol must be improved in the future to enable FE models of the full bone after implant removal. Fourth, only nine proximal femora were included in this study and there was considerable variability of implants. However, collecting a homogeneous sample of femora with a history of a fracture and treatment with a CMN is challenging, and, to the best of our knowledge, no study has yet used such specimens for validation of an FE model. The variability may also be seen as a strength of this study, as it well-resembles the clinical reality, and the FE models predicted the femoral strength accurately despite the sample heterogeneity. Finally, the influence of clinical factors (e.g. systemic disease, medication) on femoral strength could not be assessed due to limited data. Future studies should apply the presented patient-specific FE modelling approach to a larger in vivo cohort while collecting clinical data to explore their relationship with femoral strength after CMN removal.

In conclusion, CT-based FE models can pre-operatively predict femoral strength after CMN removal with high accuracy relative to ex vivo experiments. Predicted safety factors for activities of daily living were overall low, but this result may be attributed to the age of the body donors (87 years on average). Thus, pre-operative FE model predictions of femoral strength and safety factors could be useful to guide the decision on CMN removal and permissible post-operative loading particularly in younger patients.

## Electronic supplementary material

Below is the link to the electronic supplementary material.

Supplementary Material 1.

## Data Availability

The datasets generated during and/or analysed during the current study are available from the corresponding author on reasonable request.
