# Supplementary material for: Femoral strength after cephalomedullary nail removal can be predicted preoperatively using CT based FE models
Source: Sci Rep. 2025 Jun 6;15:19969. doi: 10.1038/s41598-025-02424-x (PMC12144080; doi:10.1038/s41598-025-02424-x)
Supplement: Supplementary file 1 — Supplementary Material 1. [file 41598_2025_2424_MOESM1_ESM.pdf]

Supplementary material to:

Femoral strength after cephalomedullary nail removal can be predicted preoperatively using CT based FE models

Alexander Synek[1], Gilbert M. Schwarz[2,3], Andreas G. Reisinger[1,4], Stephanie Huber[5], Sylvia Nürnberger[2], Lena Hirtler[3], Jochen G. Hofstaetter[5,6], Dieter H. Pahr[1]

[1] Institute of Lightweight Design and Structural Biomechanics, TU Wien, Vienna, Austria
[2] Department of Orthopedics and Trauma-Surgery, Division of Trauma-Surgery, Medical University of Vienna, Vienna, Austria
[3] Center for Anatomy and Cell Biology, Medical University of Vienna, Vienna, Austria.
[4] Division Biomechanics, Karl Landsteiner University of Health Sciences
[5] Michael-Ogon-Laboratory for Orthopaedic Research, Orthopedic Hospital Vienna Speising, Vienna, Austria
[6] 2nd Department, Orthopedic Hospital Vienna, Vienna, Austria

Appendix A. Effect of element size

The effect of the FE model element size on the accuracy of femoral strength predictions is discussed in the following. It is important to note that the FE modelling workflow followed a previous study by Dall'Ara et al. [8] , which was developed for FE models with an element side length of 3 mm. In this workflow, a smaller element side length will also result in a smaller volume used to infer the element-specific homogenized material properties. If this volume is excessively decreased, the homogenization assumptions may be violated, and the FE model predictions may deteriorate. Smaller volumes could also exacerbate the influence of image artefacts. On the other hand, an element side length of 3 mm may be too coarse to account for geometrical details of the previously fractured and healed femora in this study. Therefore, FE models of all nine specimens were created using 3 mm element side length (reference), as well as 2 mm and 1 mm element side length. Femoral strength predictions were then compared to experimental measurements using R^2, *CCC* and *RMSE* as shown in Table A.1. The accuracy of the FE model predictions remained almost unchanged when using 2 mm element side length, but slightly deteriorated at an element side length of 1 mm. These results highlight that the FE modelling workflow should be carefully applied as a whole and the originally proposed element size should be used. Decreasing the element size enhances geometric fidelity, but may also require changes in the material modelling approach.

Table A.1 Correlations of FE model predictions of femoral strength with the experimental measurements using different element sizes. * marks the element size of the original workflow [8] and used in this study.

Element side length in mm	R^2	*CCC*	*RMSE* in N
3*	0.94	0.97	159.88
2	0.95	0.97	151.92
1	0.89	0.87	316.49

Appendix B. Material constants and scaling

FE models using the isotropic non-linear bone material model of Dall'Ara et al. [8] achieved very good correlations for the prediction of femoral strength in the past [8] (R^2=0.8 for stance configuration), but underestimated the experimental values (regression equation: $F_{max,Exp}=1.29F_{max,FE}+2472$). This was also observed in a later study which used the same FE modelling workflow, material model and constants to predict the strength of femora with metastatic lesions [27]. In agreement with those results, a very good correlation (R^2=0.92) but an underestimation of the experimentally measured femoral strength was found in this study using the original material constants, leading to a relatively low *CCC*=0.76 and high *RMSE*=427.95 N (Table B.1).

To circumvent this limitation and provide quantitatively correct estimates for clinical interpretation, the material constants were scaled in this study. However, care was taken to keep changes to a minimum. The scaling was driven by reducing the constant E_{max}, which is the elastic modulus for a pore-less material (i.e. $\rho=1$). This constant was introduced in Dall'Ara et al. [8] to extrapolate the material properties of trabecular bone to those of cortical bone. Note that E_{max} scales both the elastic and yield properties as it is included in the extrapolation function (called "tissue function" in Dall'Ara et al. [8]). To keep the extrapolated yield strengths at $\rho=1$ unchanged, a scaling factor *s* for the trabecular bone yield strength constants for tension, compression and shear (σ_0^+ , σ_0^-, τ_0) was computed. This scaling factor can be computed as $s = E_{\max}/E_{\max}^*$, where $E_{\max}$ and $E_{\max}^*$ are the original and adapted constants, respectively.

To find the optimal constants, E_{max} was reduced from 24128 MPa (original value) to 18000 MPa in 1000 MPa steps, s was computed using the above equation to scale σ_0^+ , σ_0^-, τ_0 , and the FE models were solved for all nine specimens. As shown in Table B.1, the coefficient of determination (R^2) remained almost constant, whereas *CCC* increased and *RMSE* decreased. At E_{max}=18000 MPa, the *RMSE* started to increase again, which is why in a final iteration E_{max}=18500 MPa was chosen for this study.

Note that a large variability of the bone material constants is generally long-recognized and the adapted elastic modulus of E_{max}=18500 MPa, representing pore-less cortical bone material, is still well in line with experimental results [28].

Table B.1 Results from scaling the material constants based on E_{max}. The factor s is used to scale the trabecular bone yield strengths σ_0^+ , σ_0^-, τ_0 such that the yield strengths at ρ=1 are consistent with Dall'Ara et al. [8]. * marks the material constants used in this study.

Iteration	E_{max} in MPa	*s*	R^2	*CCC*	*RMSE* in N
Original [8]	24128	1.00	0.920	0.76	427.95
Scaling 1	23000	1.05	0.922	0.82	365.34
Scaling 2	22000	1.10	0.925	0.87	309.54
Scaling 3	21000	1.15	0.929	0.91	252.42
Scaling 4	20000	1.21	0.933	0.94	199.82
Scaling 5	19000	1.27	0.937	0.96	164.35
Scaling 6	18000	1.34	0.941	0.96	167.41
Scaling 7*	18500	1.30	0.939	0.97	159.88

Supplementary figures and tables

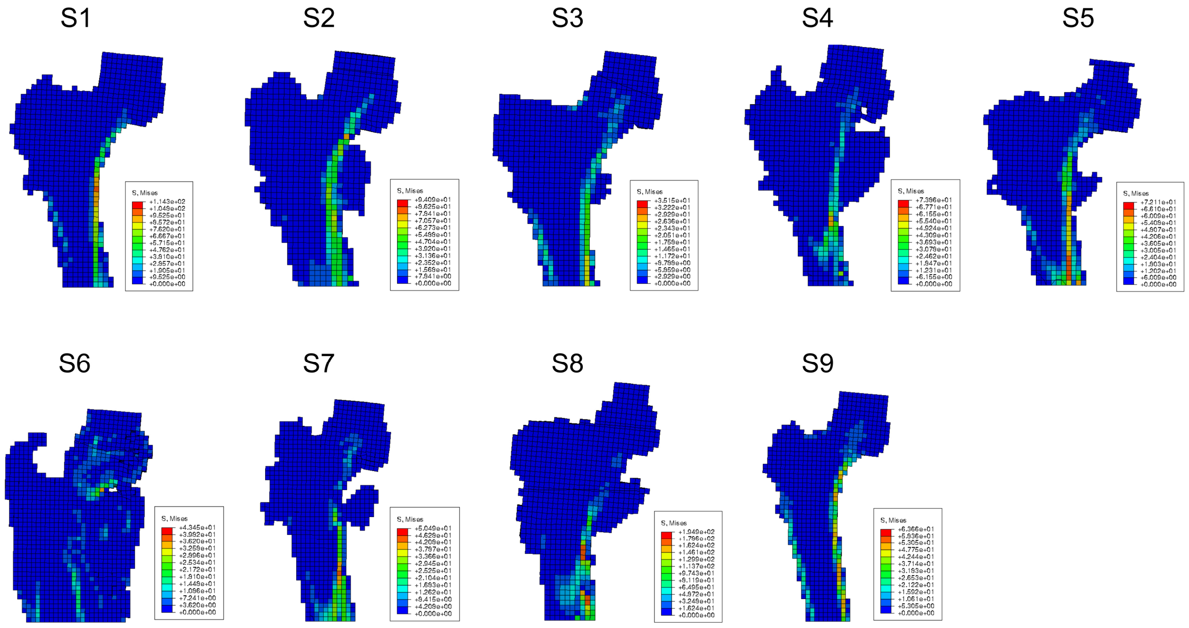

Supplementary Fig. S1 Complementary comparison of von Mises stress distributions in the FE models at the increment of peak force (i.e. at F_{max}). The colormap range was scaled to the specimen-specific peak von Mises stresses in the shown cross section. For a comparison of damaged regions, the reader is referred to Fig. 6 of the main article.

Supplementary Table S1 SF_{100} safety factors. The SF_{100} safety factors were obtained using in vivo hip joint loads of the subject with highest relative loads and scaled to 100 kg body weight from Bergmann et al. [29] ("HIGH100 Average" dataset).

Specimen	Cycling	Sit Down	Stand Up	Knee Bend	Walking	Stance	Strais Up	Stairs Down	Jogging
S1	1.71	0.73	0.56	0.68	0.75	0.64	0.60	0.55	0.44
S2	1.57	0.67	0.51	0.63	0.69	0.59	0.55	0.51	0.41
S3	0.61	0.26	0.20	0.24	0.27	0.23	0.21	0.20	0.16
S4	1.30	0.56	0.42	0.52	0.57	0.49	0.45	0.42	0.34
S5	1.34	0.57	0.44	0.54	0.59	0.50	0.47	0.44	0.35
S6	1.45	0.62	0.47	0.58	0.63	0.55	0.51	0.47	0.38
S7	1.11	0.48	0.36	0.44	0.48	0.42	0.39	0.36	0.29
S8	2.38	1.02	0.78	0.95	1.04	0.90	0.83	0.77	0.62
S9	1.84	0.79	0.60	0.73	0.80	0.69	0.64	0.59	0.48
